# Supplementary material for: Bi-Allelic MARVELD2 Variant Identified with Exome Sequencing in a Consanguineous Multiplex Ghanaian Family Segregating Non-Syndromic Hearing Loss
Source: Int J Mol Sci. 2025 Apr 3;26(7):3337. doi: 10.3390/ijms26073337 (PMC11989440; doi:10.3390/ijms26073337)
Supplement: Supplementary file 1 [file ijms-26-03337-s001.zip › ijms-3532175-supplementary.pdf]

Table S1. Distribution of previously reported M*ARVELD2* variants associated with non-syndromic hearing loss.

Number	Variant	HGVS	Type of mutation	Family Origin/ population	Evidence of consanguinity	Pattern of inheritance	Type of hearing impairment	Reference
**1**	c.1058dupT	p.Val354SerfsTerfs*5	Nonsense	Ghana	YES	AR	NS	This study
**2**	c.1331+1G > A	IVS4+1G > A	Splice site	Iran	YES	AR	NS	[28]
**3**	c.1543delA	Lys517ArgTerfs*18	Deletion	Iran	NO	AR	NS	[29]
**4**	c.1331+2 T > C	IVS4+2T > C	Splice site	Roma, Czech Republic	YES	AR	NS	[30]
**5**	c.1331+2T > C	c.1331+2T > C	Splice site	Switzerland	NO	AR	S	[31]
**6**	c.1331+2T > C	c.1331+2T > C	Splice site	Hungary	NO	AR	NS	[32]
**7**	c.1331+1G > A	IVS4+1G > A	Splice site	Pakistan	YES	AR	NS	[33]
**8**	c.1331+2 T > C	IVS4+2T > C	Splice site	Pakistan	YES	AR	NS	[34]
**9**	c.1331+2 T > C	IVS4+2T > C	Splice site	Slovakia	NO	AR	NS	[35]
**10**	c.1331+2 T > C	IVS4+2T > C	Splice site	Hungary	NO	AR	NS	[35]
**11**	c.1183-1G > A	IVS3-1G > A	Splice site	Pakistan	YES	AR	NS	[36]
**12**	IVS4+2delTGAG	IVS4+2delTGAG	Deletion	Pakistan	YES	AR	NS	[36]
**13**	c.1331+2 T > C	IVS4 + 2T > C	Splice site	Pakistan	YES	AR	NS	[36]
**14**	c.1498C > T	p.Arg500X	Nonsense	Pakistan	YES	AR	NS	[36]
**15**	c.1331 + 2T > C	IVS4 + 2T > C	Splice site	Pakistan	YES	AR	NS	[37]
**16**	c.1183 - 1G > A	IVS3 - 1G > A	Splice site	Pakistan	YES	AR	NS	[37]
**17**	c.1331 + 2T > C	IVS4 + 2T > C	Splice site	Slovakia	YES	AR	S	[37]
**18**	c.1331 + 2T > C	IVS4 + 2T > C	Splice site	India	NO	AR	NS	[38]
**19**	c.1208_1211del	p.Arg403Lysfs*11	Splice site	India	N/A	AD	NS	[39]
**20**	c.1555delinsAA	p.Asp519Lysfs*12	Nonsense	Iran	YES	AR	NS	[32]
**21**	c.1006C > T	p.Arg336Trp	Missense	Chinese	N/A	AR	NS	[40]
**22**	c.730G > A	p.Gly244Arg	Missense	Chinese	N/A	AR	NS	[40]
**23**	c.772G > A	p.Val258Met	Missense	Chinese	N/A	AR	NS	[40]
**24**	c.949C > G	p.Arg317Gly	Missense	Chinese	N/A	AR	NS	[40]

AR = Autosomal recessive; AD = Autosomal dominant; HGVS = Human Genome Variation Society; NS = Non-syndromic; S = Syndromic; N/A = Not available; A = Adenine; G = Guanine; C = Cytosine; and T = Thymine

Table S2. Western blot Densitometric Analysis

Experiment	MARVELD2	β-Actin	HK Protein Normalization	Relative Expression
Wild Type	27324,238	24012,652	1,137910048	1
Mutant	1962,891	24106,409	0,081426105	0,071557594

Table S3. HEK-293 glass bottom dish culture transfection reagents composition

Test	DMEM (µL)	XtremeGene HP Reagent (µL)	Plasmid DNA (2.0 µg) / µL
Wild-Type	191.98	6.00	2.02
Mutant	190.28	6.00	3.72
No DNA	97.00	3.00	-

DMEM = Dulbecco's Modified Eagle Medium

Table S4. HEK-293 cells cover slip culture transfection reagents composition

Test	DMEM (µL)	XtremeGene HP Reagent (µL)	Plasmid DNA (4.0 µg) / µL
Wild-Type	383.96	12.00	4.04
Mutant	380.78	12.00	7.22
No DNA	97.00	3.00	-

DMEM = Dulbecco's Modified Eagle Medium

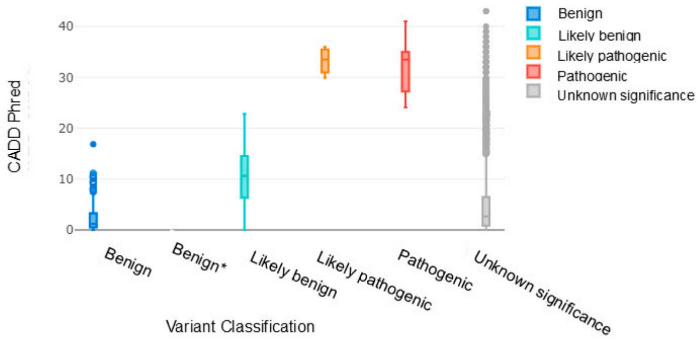

Figure S1. Combined Annotation Dependent Depletion (CADD) Phred scores against MARVELD2 reported variant classification. Figure retrieved from ClinVar (https://www.ncbi.nlm.nih.gov/clinvar/) accessed on 23 July 2023.

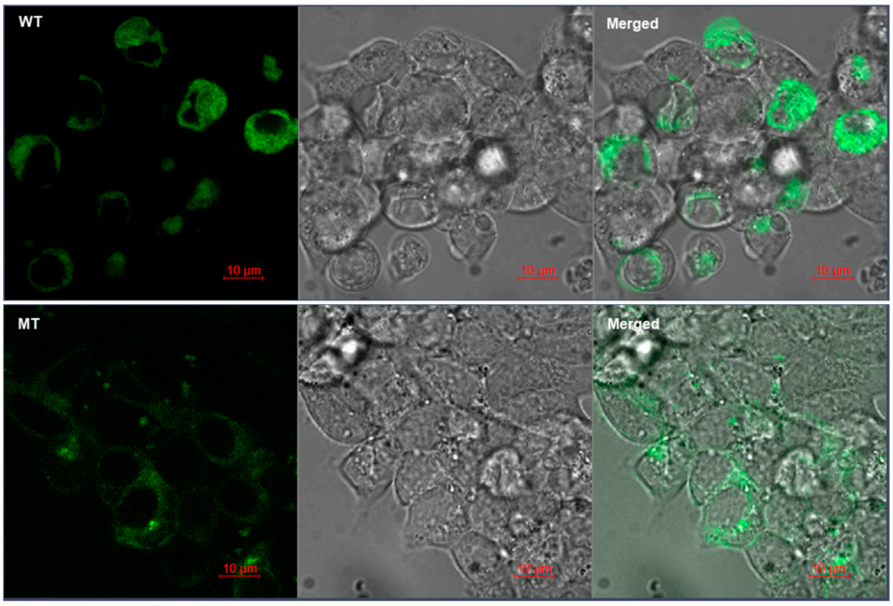

Figure S2. Mutant MARVELD2 Fluorescence and immunocytochemistry. HEK 293 Cells 24- and 48-hours post-transfection confocal imaging. Panel A) Wild type and Mutant EYFP expression. Panel B) Wild type and Mutant EYFP localization Bars = 10 μm

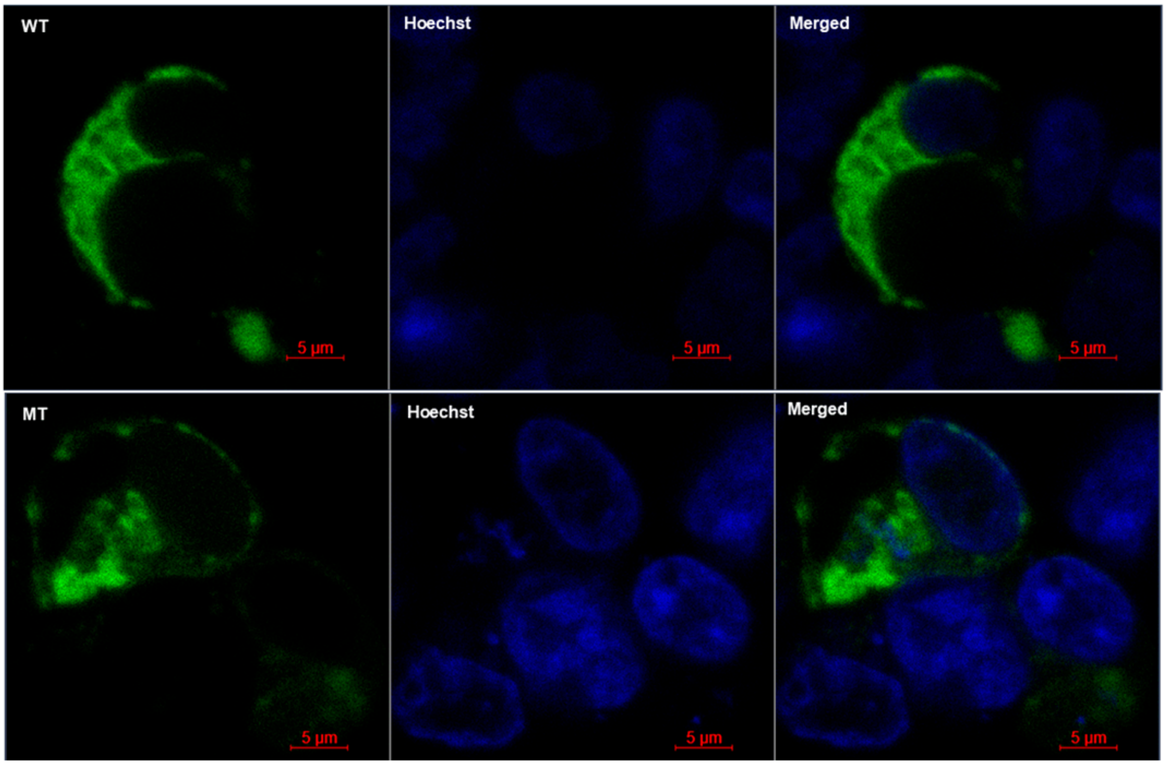

Figure S3. Mutant MARVELD2 Immunocytochemistry. Panel A) Wild type and Mutant EYFP expression. Panel B) Wild type and Mutant EYFP localization. Bars = 5 µm

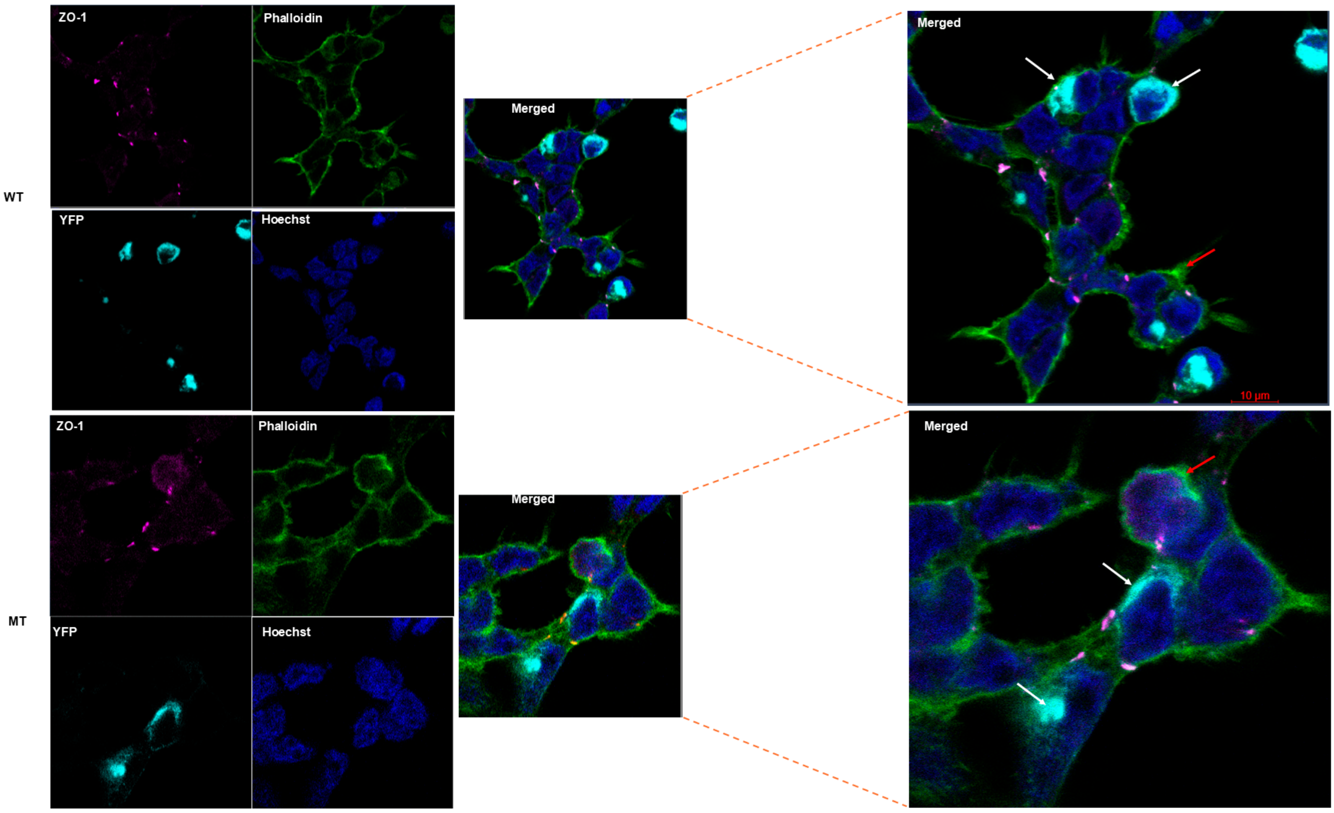

Figure S4. Wild type or Mutant MARVELD2-N-EYFP, zonula occluden-1 (ZO-1), and phalloidin staining along cellular margins and tricellular junctions. HEK-293 cells 48 hours post-transfection localization of MARVELD2-p.Val354SerfsTerfs*5 protein (WT or MT) were stained for ZO-1 and phalloidin, and viewed using confocal microscopy. MARVELD2 proteins were viewed using YFP

channel, ZO-1 by Texas-red (depicted as violet), phalloidin actin stain using Oregon green (green) and Hoechst stain for DNA in the nuclei (blue). Wild-type Panel). Wild-type transfected HEK-293 cells; localized expressed MARVELD2-N-EYFP is condensed and consistent (EYFP panel and white arrows in merged panel). Phalloidin F-actin stained; accumulation demonstrated by observed positive-spots along cell margins. Mutant Panel). In mutant transfected HEK-293 cells, localization of expressed tagged MARVELD2-p.Val354SerfsTerfs*5 seems to be diffused (EYFP panel). Phalloidin F-actin stained; accumulation also revealing relatively less stress fibres distribution seen as positive spots along membrane barrier compared to the wild type. Bars = 10 μm.

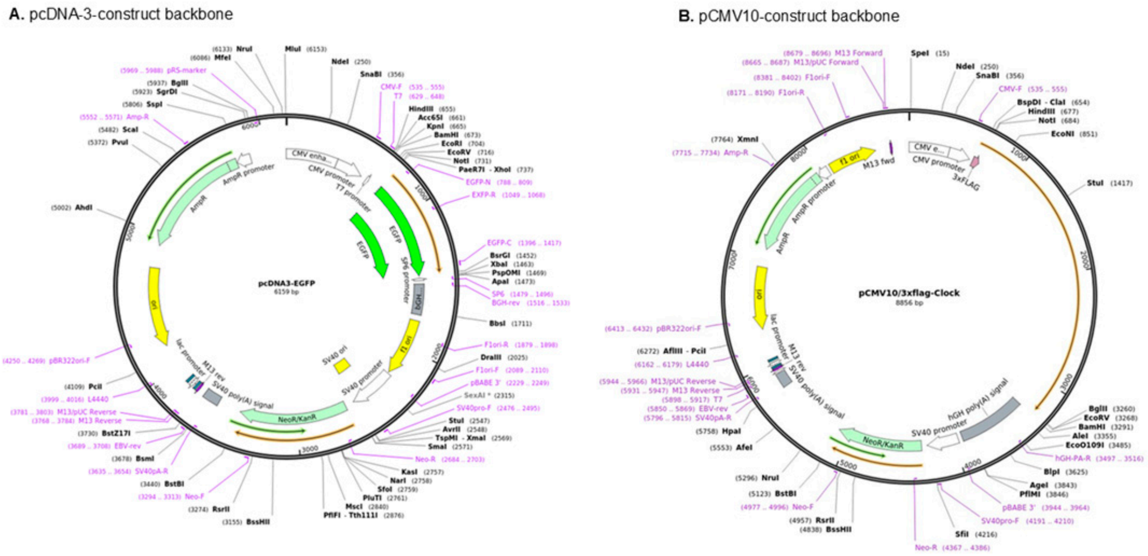

Figure S5. pCMV10 and pcDNA-3 constructs backbone and components.

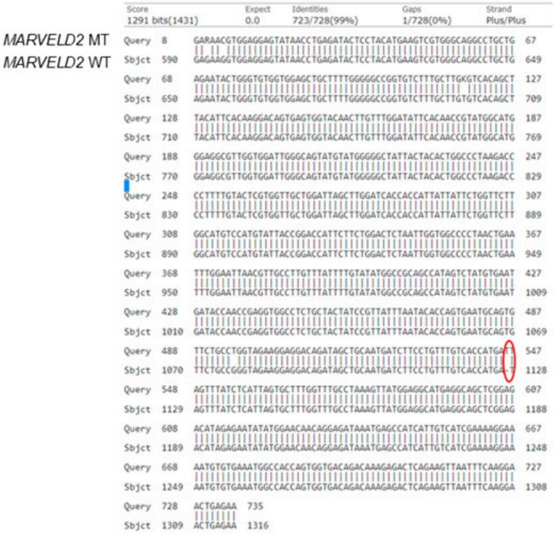

Figure S6. Sanger validation of site-directed mutagenesis; thymine (T) duplication circled in red.
